# Mechanical Thrombectomy for Acute Ischemic Stroke in Nonagenarians: A Dilemma in Treatment Approach

**DOI:** 10.7759/cureus.75563

**Published:** 2024-12-11

**Authors:** Subash Phuyal, Alamelu Alagappan, Prakash Phuyal, Biswajit Sahoo, Manoj Kumar Nayak

**Affiliations:** 1 Neuroimaging and Interventional Neuroradiology, Upendra Devkota Memorial National Institute of Neurological and Allied Sciences, Kathmandu, NPL; 2 Radiodiagnosis, All India Institute of Medical Sciences, Bhubaneswar, Bhubaneswar, IND

**Keywords:** ais (acute ischemic stroke), elderly mechanical thrombectomy, elderly stroke, major arterial occlusion, nonagenarians

## Abstract

Stroke is one of the major causes of mortality and morbidity, particularly among the elderly population. As the general population ages, cerebrovascular disease is anticipated to increase in prevalence. Strokes can manifest as either hemorrhagic or ischemic events. While mechanical thrombectomy is efficacious in the treatment of major arterial occlusion, many studies have excluded nonagenarians due to anticipated poor functional outcomes. The functional prognosis in nonagenarians is influenced by various factors, including procedural challenges related to vessel tortuosity, collateral circulation status, and multiple medical comorbidities. Herein, we report a case of acute ischemic stroke (AIS) in a nonagenarian male with major arterial occlusion successfully managed via mechanical thrombectomy, resulting in an excellent post-procedural outcome at the three-month follow-up.

## Introduction

Stroke can manifest as either hemorrhagic or ischemic events. In cases of acute ischemic stroke (AIS) with large arterial occlusion, mechanical thrombectomy (MT) has emerged as an effective procedure for recanalizing occluded vessels [1-3]. However, nonagenarians have not been adequately represented or eliminated from several randomized clinical trials, creating a dilemma regarding the effectiveness of MT in older adults [4]. Although age is not regarded as a contra-indication for MT, recent landmark MT trials have not provided clear therapy recommendations for patients over 90 years old [5]. Given the higher comorbidities and potential for poorer prognosis in patients over 90 compared to younger patients, it is crucial to evaluate the effectiveness of MT in nonagenarians. In this article, we report a nonagenarian with acute distal M1 segment of middle cerebral artery (MCA) occlusion, successfully recanalized using contact aspiration thrombectomy, resulting in a favorable post-procedure outcome.

## Case presentation

**Figure 1:**
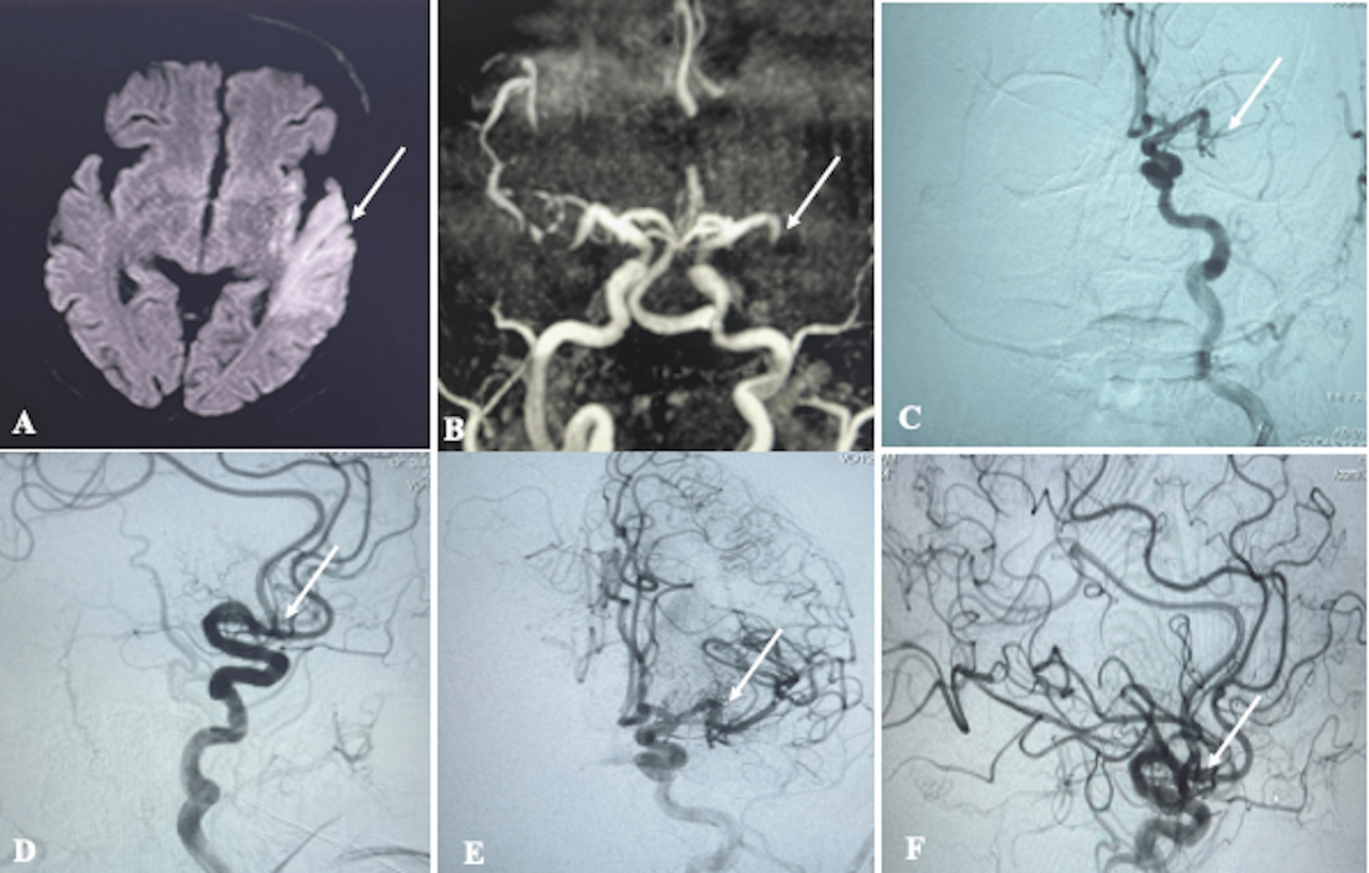
Magnetic resonance imaging (MRI), digital subtraction angiography (DSA) images of the patient with stroke. Axial diffusion-weighted imaging (DWI) (A) showed acute infarct (white arrow) in the left middle cerebral artery (MCA) territory. Time of flight (TOF) image (B) showed left-MCA complete cut-off (white arrow). DSA images (AP in image C, lateral in image D) showed left distal MCA cutoff (white arrows). Post-procedure DSA images (AP in image E, lateral in image F) showed complete recanalization of left distal MCA with filling of the distal branches (white arrows).

A 95-year-old gentleman presented with sudden onset slurring of speech, dysarthria, and facial deviation with right hemiparesis for five hours. He was a known diabetic and hypertensive, taking regular medication for 20 years. No history of similar illness was present before. His National Institute of Health Stroke Scale (NIHSS) was 11, and his modified Rankin scale (mRS) was 4. Magnetic resonance imaging (MRI) showed acute infarct in the left posterior temporal lobe with an Alberta stroke program early CT (ASPECT) score of 7 (Figure 1A).

Time of flight (TOF) magnetic resonance angiography (MRA) showed occlusion of the left distal M1 MCA (Figure 1B). Following informed consent, a triaxial approach was employed for contact aspiration thrombectomy. A 6F Neuron Max (088) (Penumbra, Alameda, CA, USA) was placed in the left proximal cervical internal carotid artery through right femoral access, the diagnostic angiogram, revealed a complete occlusion of proximal left MCA (Figure 1C, 1D) The ACE 68 reperfusion catheter (Penumbra) with a large bore aspiration was inserted into the guiding sheath (Neuron Max) via a 035′′ (150 cm) Terumo guidewire (Terumo, Tokyo, Japan). The ACE 68 aspiration catheter and Headway 27 microcatheter/Traxcess 14 microguidewire assembly (Microvention, Alameda, CA, USA) were advanced into the occluded left MCA. The ACE 68 catheter was navigated up to the clot’s level using a microcatheter/microguidewire assembly. Thrombus aspiration was carried out utilizing a penumbra aspiration pump in combination with an ACE 68 catheter. The occluded MCA was recanalized after a single pass with TICI (thrombolysis in cerebral infarction) 3 recanalization (Figure 1E, 1F). Post-procedure, the patient experienced a complete improvement in speech and mild weakness in right hemiparesis (4/5). At the follow-up on the 90th day, his mRS score was 0, indicating an excellent functional outcome.

## Discussion

Most research indicates that older stroke patients face a higher risk of poorer clinical outcomes compared to younger patients [6,7]. Among octogenarians, combining mechanical thrombectomy (MT) with medical management is considered superior to intravenous thrombolysis (IVT) alone. The most robust evidence regarding acute ischemic stroke's endovascular management comes from the HERMES collaborator meta-analysis, which includes a subgroup analysis of older patients [8]. This analysis suggests that MT benefits patients aged eighty or older. However, the population of extremely elderly patients (aged ≥90) in this analysis was statistically insignificant (0.8%; 5 out of 634) [8]. However, the HERMES trial correlated between older age and functional dependence in both the intervention and control groups despite advancements in intervention in the elderly population [8]. Consequently, treatment recommendations based on the HERMES trial may apply primarily to octogenarians and may not fully extend to nonagenarians and centenarians [8]. Duffis et al., in their meta-analysis, showed a significantly higher incidence of mortality, functional dependence, and symptomatic hemorrhage in patients aged >80 years compared to the young population [9]. Sussman et al., in their study, compared the outcomes between nonagenarians and octagenarians and showed that nonagenarians might be at higher risk of symptomatic intracranial hemorrhage than octagenarians despite similar stroke and treatment-related factors [10]. While earlier studies highlighted older age as a significant factor of unfavorable outcomes after endovascular therapy, subgroup analyses from recent trials suggest that this may not be true with the recently developed recanalization devices [8]. Moreover, studies have shown comparable recanalization rates between older and younger patients, although some data indicate a downward trend among the elderly [9,11-13]. This could be attributed to the increased tortuosity of cerebral vasculature with age, posing technical challenges and potentially leading to incomplete recanalization [9]. Additionally, older patients may have poorer collateral blood supply, resulting in early tissue loss and poorer outcomes [14]. Associated white matter disease has also emerged as a separate factor for determining the outcomes in patients undertaking endovascular management for major arterial occlusion [15]. Furthermore, older patients often have more medical comorbidities, which can influence their functional recovery following a stroke [16].

## Conclusions

Although a significantly higher incidence of mortality, functional dependence, and symptomatic hemorrhage treated with mechanical thrombectomy (MT) for major arterial occlusion in patients aged>80 years described in the literature, MT can be a safe and efficacious tool for vessel recanalization, leading to improved functional outcomes. However, achieving optimal outcomes hinges on procedural time influenced by vessel tortuosity, collateral status, and medical comorbidities. Further large-scale clinical trials are warranted to solidify the evidence supporting the efficacy of MT, specifically in nonagenarians.
